# Achieving the Recommended Endotracheal Tube Cuff Pressure: A Randomized Control Study Comparing Loss of Resistance Syringe to Pilot Balloon Palpation

**DOI:** 10.1155/2017/2032748

**Published:** 2017-12-31

**Authors:** Fred Bulamba, Andrew Kintu, Nodreen Ayupo, Charles Kojjo, Lameck Ssemogerere, Agnes Wabule, Arthur Kwizera

**Affiliations:** ^1^Department of Anaesthesia and Critical Care, Faculty of Health Sciences, Busitema University, Mbale, Uganda; ^2^Department of Anaesthesia and Critical Care, College of Health Sciences, Makerere University, Kampala, Uganda

## Abstract

**Background:**

Both under- and overinflation of endotracheal tube cuffs can result in significant harm to the patient. The optimal technique for establishing and maintaining safe cuff pressures (20–30 cmH_2_O) is the cuff pressure manometer, but this is not widely available, especially in resource-limited settings where its use is limited by cost of acquisition and maintenance. Therefore, anesthesia providers commonly rely on subjective methods to estimate safe endotracheal cuff pressure. This study set out to determine the efficacy of the loss of resistance syringe method at estimating endotracheal cuff pressures.

**Methods:**

This was a randomized clinical trial. We enrolled adult patients scheduled to undergo general anesthesia for elective surgery at Mulago Hospital, Uganda. Study participants were randomized to have their endotracheal cuff pressures estimated by either loss of resistance syringe or pilot balloon palpation. The pressures measured were recorded.

**Results:**

One hundred seventy-eight patients were analyzed. 66.3% (59/89) of patients in the loss of resistance group had cuff pressures in the recommended range compared with 22.5% (20/89) from the pilot balloon palpation method. This was statistically significant.

**Conclusion:**

The loss of resistance syringe method was superior to pilot balloon palpation at administering pressures in the recommended range. This method provides a viable option to cuff inflation.

## 1. Background

High-volume low-pressure cuffed endotracheal tubes (ETT) are the standard of airway protection. However, they have potential complications [1–3]. Underinflation increases the risk of air leakage and aspiration of gastric and oral pharyngeal secretions [4, 5]. Cuff pressures less than 20 cmH_2_O have been shown to predispose to aspiration which is still a major cause of morbidity, mortality, length of stay, and cost of hospital care as revealed by the NAP4 UK study. In this cohort, aspiration had the second highest incidence of primary airway-related serious events [6].

On the other hand, overinflation may cause catastrophic complications. It has been demonstrated that, beyond 50 cmH_2_O, there is total obstruction to blood flow to the tracheal tissues. This has been shown to cause severe tracheal lesions and morbidity [7, 8]. However, less serious complications like dysphagia, hoarseness, and sore throat are more prevalent [9–11]. There are a number of strategies that have been developed to decrease the risk of aspiration, but the most important of all is continuous control of cuff pressures.

To achieve the optimal ETT cuff pressure of 20–30 cmH_2_O [3, 8, 12–14], ETT cuffs should be inflated with a cuff manometer [15, 16]. However, these are prohibitively expensive to acquire and maintain in many operating theaters, and as such, many anesthesia providers resort to subjective methods like pilot balloon palpation (PBP) which is ineffective [1, 2, 16–20]. Alternative, cheaper methods like the minimum leak test that require no special equipment have produced inconsistent results.

A newer method, the passive release technique, although with limitations, has been shown to estimate cuff pressures better [21–24]. This method has been achieved with a modified epidural pulsator syringe [13, 18], a 20 ml disposable syringe, and more recently, a loss of resistance (LOR) syringe [21, 23, 24]. Compared with the cuff manometer, it would be cheaper to acquire and maintain a loss of resistance syringe especially in low-resource settings.

Our study set out to investigate the efficacy of the loss of resistance syringe in a surgical population under general anesthesia. ETT cuff pressures would be measured with a cuff manometer following estimation by either the PBP method or the LOR method. Our secondary objective was to determine the incidence of postextubation airway complaints in patients who had cuff pressures adjusted to 20–30 cmH_2_O range or 31–40 cmH_2_O range.

## 2. Materials and Methods

### 2.1. Study Design and Setting

This single-blinded, parallel-group, randomized control study was performed at Mulago National Referral Hospital, Uganda. The hospital has a bed capacity of 1500 inpatient beds, 16 operating rooms, and a mean daily output of 90 surgical operations. Anesthesia services are provided by different levels of providers including physician anesthetists (anesthesiologists), residents, and nonphysician anesthetists (anesthetic officers and anesthetic officer students). In the early years of training, all trainees provide anesthesia under direct supervision. In the later years, however, they can administer anesthesia either independently or under remote supervision.

### 2.2. Study Population

We included ASA class I to III adult patients scheduled to receive general anesthesia with endotracheal intubation for elective surgical operation. All patients with any of the following conditions were excluded: known or anticipated laryngeal tracheal abnormalities or airway trauma, preexisting airway symptoms, laparoscopic and maxillofacial surgery patients, and those expected to remain intubated beyond the operative room period.

### 2.3. Randomization, Blinding, and Enrollment

After screening, participants were allocated to either the PBP or the LOR group using block randomization, achieving a 1 : 1 allocation ratio. The allocation sequence was generated by an Internet-based application with the following input: nine sets of unsorted sequences, each containing twenty unique allocation numbers (1–20). Numbers 1–10 were labeled LOR, and numbers 11–20 were labeled PBP. The allocation sequence was concealed from the investigator by inserting it into opaque envelopes (according to the clocks) until the time of the intervention.

At the time of the intervention, the study investigator retrieved the next available envelope, which indicated the intervention group, from the next available block envelope and handed it to the research assistant. The patient was the only person blinded to the intervention group.

The anesthesia providers were either physician anesthetists (anesthesiologists or residents) or nonphysicians (anesthetic officer or anesthetic officer student). Students were under the supervision of a senior anesthetic officer or an anesthesiologist.

### 2.4. Sample Size

To detect a 15% difference between PBP and LOR groups, it was calculated that at least 172 patients would be required to be 80% certain that the limits of a 95%, two-sided interval included the difference.

### 2.5. Intervention and Anesthetic Technique

Every patient was wheeled into the operating theater and transferred to the operating table. Basic routine monitors were attached as per hospital standards. A wide-bore intravenous cannula (16- or 18-G) was placed for administration of drugs and fluids.

The patient was then preoxygenated with 100% oxygen and general anesthesia induced with a combination of drugs selected by the anesthesia care provider. These included an intravenous induction agent, an opioid, and a muscle relaxant. All patients received either suxamethonium (2 mg/kg, max 100 mg to aid laryngoscopy) or cisatracurium (0.15 mg/kg at for prolonged muscle relaxation) and were given optimal time before intubation. Using a laryngoscope, tracheal intubation was performed, ETT position confirmed, and secured with tape within 2 min. The size of ETT (POLYMED Medicure, India) was selected by the anesthesia care provider. With the patient's head in a neutral position, the anesthesia care provider inflated the ETT cuff with air using a 10 ml syringe (BD Discardit II). A research assistant (different from the anesthesia care provider) read out the patient's group, and one of the following procedures was followed.

  PBP group (active comparator): in this group, the anesthesia care provider was asked to reduce or increase the pressure in the ETT cuff by inflating with air or deflating the pilot balloon using a 10 ml syringe (BD Discardit II) while simultaneously palpating the pilot balloon until a point he or she felt was appropriate for the patient. When this point was reached, the 10 ml syringe was then detached from the pilot balloon, and a cuff manometer (VBM, Medicintechnik Germany. Accuracy −2 cmH_2_O) was attached. The pressure reading of the VBM was recorded by the research assistant.
  LOR group (experimental): in this group, the research assistant attached a 7 ml plastic, luer slip loss of resistance syringe (BD Epilor, USA) containing air onto the pilot balloon. The cuff was then briefly overinflated through the pilot balloon, and the loss of resistance syringe plunger was allowed to passively draw back until it ceased. This point was observed by the research assistant and witnessed by the anesthesia care provider. The loss of resistance syringe was then detached, the VBM manometer was attached, and the pressure reading was recorded.
  Cuff pressure adjustment: in both arms, very high and very low pressures were adjusted as per the recommendation by the ethics committee. In case of a very low pressure reading (below 20 cmH_2_O), the ETT cuff pressure would be adjusted to 24 cmH_2_O using the manometer. On the other hand, high cuff pressures beyond 50 cmH_2_O were reduced to 40 cmH_2_O.

Also, at the end of the pressure measurement in both groups, the manometer was detached, breathing circuit was attached to the ETT, and ventilation was started. The patient was maintained on isoflurane (1–1.8%) mixed with 100% oxygen flowing at 2 L/min. Anesthesia continued without further adjustment of ETT cuff pressure until the end of the case. Nitrous oxide and medical air were not used as these agents are unavailable at this hospital. All patients who received nondepolarizing muscle relaxants were reversed with neostigmine 0.03 mg/kg and atropine 0.01 mg/kg at the end of surgery.

### 2.6. Primary Outcome

Cuff pressure reading of the VBM manometer was recorded by the research assistant. The individual anesthesia care providers participated more than once during the study period of seven months.

### 2.7. Secondary Outcome

The patients were followed up and interviewed only once at 24 hours after intubation for presence of cough, sore throat, dysphagia, and/or dysphonia. Only two of the four research assistants reviewed the patients postoperatively, and these were blinded to the intervention arm. This outcome was compared between patients with cuff pressures from 20 to 30 cmH_2_O range and those from 31 to 40 cmH_2_O following the initial correction of cuff pressures.

### 2.8. Data Management

All data were double entered into EpiData version 3.1 software (The EpiData Association, Odense, Denmark), with range, consistency, and validation checks embedded to aid data cleaning. The data were exported to and analyzed using STATA software version 12 (StataCorp Inc., Texas, USA).

Categorical data are presented in tabular, graphical, and text forms and categorized into PBP and LOR groups. The chi-square test was used for categorical data. Continuous data are presented as the mean with standard deviation and were compared between the groups using the *t*-test to detect any significant statistical differences.

The magnitude of effect on the primary outcome was computed for 95% CI using the *t*-test for difference in group means. An intention-to-treat analysis method was used, and the main outcome of interest was the proportion of cuff pressures in the range 20–30 cmH_2_O in each group.

For the secondary outcome, incidence of complaints was calculated for those with cuff pressures from 20 to 30 cmH_2_O range and those from 31 to 40 cmH_2_O.

### 2.9. Data Safety Management Board

The Data Safety Management Board (DSMB) comprised an anesthesiologist, a statistician, and a member of the SOMREC IRB who would be informed of any adverse event. The study would be discontinued if 5% of study subjects in one study group experienced an adverse event associated with the study interventions as determined by the DSMB, or if a *p* value of <0.001 was obtained on an interim analysis performed halfway through patient accrual. None of these was met at interim analysis.

### 2.10. Ethical Considerations

The study was approved by the School of Medicine Research and Ethics Committee, Makerere University, and registered with http://www.clinicaltrials.gov (NCT02294422). All patients provided informed, written consent before the start of surgery.

## 3. Results

### 3.1. Baseline Characteristics

**Figure 1:**
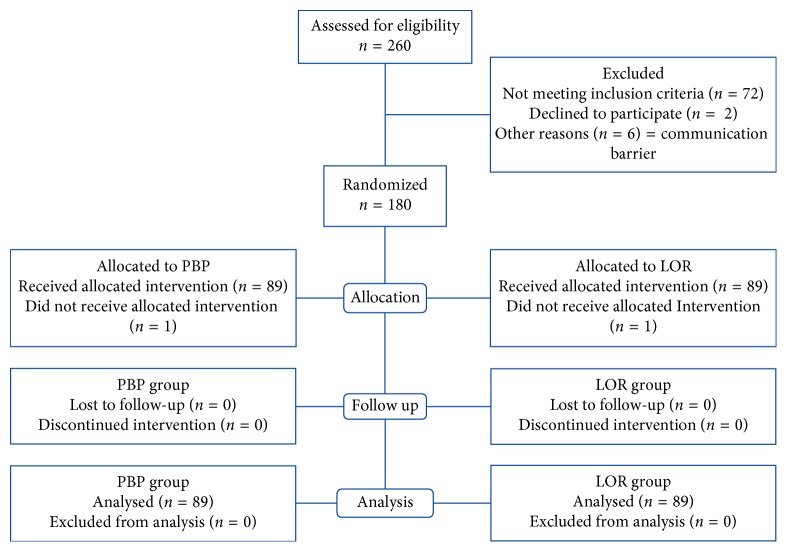
A CONSORT flow diagram of study patients. LOR = loss of resistance syringe method; PBP = pilot balloon palpation method.

A total of 178 patients were enrolled from August 2014 to February 2015 with an equal distribution between arms as shown in the CONSORT diagram in Figure 1.

The study comprised more female patients (76.4%). In addition, most patients were below 50 years (76.4%). The study groups were similar in relation to sex, age, and ETT size (Table 1).

### 3.2. ETT Cuff Pressures

Generally, the proportion of ETT cuffs inflated to the recommended pressure was less in the PBP group at 22.5% (20/89) compared with the LOR group at 66.3% (59/89) with a statistically significant positive mean difference of 0.47 with *p* value < 0.01 (0.343–0.602). Also to note, most cuffs in the PBP group were inflated to a pressure that exceeded the recommended range in the PBP group, and 51% of the cuff pressures attained had to be adjusted compared with only 12% in the LOR group (Table 2).

**Figure 2:**
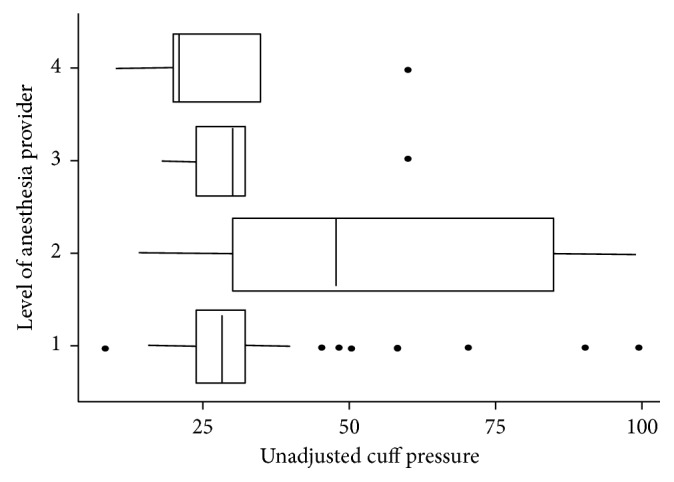
The distribution of cuff pressures achieved by the different levels of providers. 1: anesthesia resident; 2: anesthesia officer; 3: anesthesia officer student; 4: anesthesiologist.

The distribution of cuff pressures (unadjusted) achieved by the different care providers is shown in Figure 2. The difference in the number of intubations performed by the different level of providers is huge with anesthesia residents and anesthetic officers performing almost all intubation and initial cuff pressure estimations.

### 3.3. Postextubation Airway Symptoms

The total number of patients who experienced at least one postextubation airway symptom was 113, accounting for 63.5% of all patients. The incidence of postextubation airway complaints after 24 hours was lower in patients with a cuff pressure adjusted to the 20–30 cmH_2_O range, 57.1% (56/98), compared with those whose cuff pressure was adjusted to the 30–40 cmH_2_O range, 71.3% (57/80). This however was not statistically significant (*p* value 0.053) (Table 3).

## 4. Discussion

### 4.1. Study Population

We conducted a single-blinded randomized control study to evaluate the LOR syringe method in accordance with the CONSORT guideline (CONSORT checklist provided as Supplementary Materials available here).

At the study hospital, there are more females undergoing elective surgery under general anesthesia compared with males. In addition, over 90% of anesthesia care at this hospital was provided by anesthetic officers and anesthesia residents during the study period. Anesthetic officers provide over 80% of anesthetics in Uganda.

### 4.2. ETT Cuff Pressure

The primary outcome of the study was to determine the proportion of cuff pressures in the optimal range from either group. The initial, unadjusted cuff pressures from either method were used for this outcome. When considering this primary outcome, the LOR syringe method had a significantly higher proportion compared to the PBP method. This adds to the growing evidence to support the use of the LOR syringe for ETT cuff pressure estimation. There are data regarding the use of the LOR syringe method for administering ETT cuff pressures [21, 23, 24], but studies on a perioperative population are scanty. In our study, 66.3% of ETT cuff pressures estimated by the LOR syringe method were in the optimal range. Kim and coworkers, who evaluated this method in the emergency department, found an even higher percentage of cuff pressures in the “normal range” (22–32 cmH_2_O) in their study. Another study, using nonhuman tracheal models and a wider range (15–30 cmH_2_O) as the optimal, had all cuff pressures within the optimal range [21]. It is however difficult to extrapolate these results to the human population since the risk of aspiration of gastric contents is zero while working with models when compared with patients.

There is a relatively small risk of getting ETT cuff pressures less than 30 cmH_2_O with the use of the LOR syringe method [23, 24], 12.4% from the current study. Secondly, this method is still provider-dependent as they decide when plunger drawback has ceased. Precaution was taken to avoid premature detachment of the loss of resistance syringe in this study.

The PBP method, although commonly employed in operating rooms, has been repetitively shown to administer cuff pressures out of the optimal range (20–30 cmH_2_O) [2, 3, 25]. Findings from this study were in agreement, with 25.3% of cuff pressures in the optimal range after estimation by the PBP method. It should however be noted that some of these studies have been carried out in different environments (emergency rooms) and on different kinds of patients (emergency patients) by providers of varying experience [2].

Another viable argument is to employ a more pragmatic solution to prevent overly high cuff pressures by inflating the cuff until no air leak is detected by auscultation. This method is cheap and reproducible and is likely to estimate cuff pressures around the normal range. Perhaps the LOR syringe method needs to be evaluated against the “no air leak on auscultation” method.

This study shows that the LOR syringe method is better at estimating cuff pressures in the optimal range when compared with the PBP method but still falls short in comparison to the cuff manometer. In low- and middle-income countries, the cost of acquiring ($ 250–300) and maintaining a cuff manometer is still prohibitive.

### 4.3. Postextubation Airway Complaints

The secondary objective of the study evaluated airway complaints in those who had cuff pressure in the optimal range (20–30 cmH_2_O) and those above the range (31–40 cmH_2_O). The difference in the incidence of sore throat and dysphonia was statistically significant, while that for cough and dysphagia was not. The overall trend suggests an increase in the incidence of postextubation airway complaints in patients whose cuff pressures were corrected to 31–40 cmH_2_O compared with those corrected to 20–30 cmH_2_O. This however was not statistically significant (*p* value 0.052).

Considering that this was a secondary outcome, it is possible that the sample size was small, hence leading to underestimation of the incidence of postextubation airway complaints between the groups. It is however possible that these results have a clinical significance.

The complaints sought in this study included sore throat, dysphagia, dysphonia, and cough. These were adopted from a review on postoperative airway problems [26] and were defined as follows: sore throat, continuous throat pain (which could be mild, moderate, or severe), dysphagia, uncoordinated swallowing or inability to swallow or eat, dysphonia, hoarseness or voice changes, and cough (identified by a discomforting, dry irritation in the upper airway leading to a cough). All these symptoms were of a new onset following extubation.

There is consensus that keeping ETT cuff pressures low decreases the incidence of postextubation airway complaints [11].

Previous studies have shown that the incidence of postextubation airway symptoms varies from 15% to 94% in various study populations [7, 9, 11, 27] and could be affected by the method of interview employed, such as the one used in our study (yes/no questions). This study was not powered to evaluate associated factors, but there are suggestions that the levels of anesthesia providers with varying skill set and technique at direct laryngoscopy may be associated with a high incidence of complications. However, this could be a site-specific outcome.

## 5. Conclusion

Although this was a single-blinded, single-centre study, results suggest that the LOR syringe method was superior to PBP at administering pressures in the optimal range.

The high incidence of postextubation airway complaints in this study is most likely a site-specific problem but one that other resource-limited settings might identify with.

We recommend the use of the cuff manometer whenever available and the LOR method as a viable option. Alternatively, cheaper, reproducible methods, like the minimum leak test that limit overly high cuff pressures should be sought and evaluated.

## Figures and Tables

**Table 1:** Baseline characteristics of patients.

Variable	Pilot balloon palpation (*n* = 89)	Loss of resistance (*n* = 89)	Total *N* = 178	*p* value
*Age group (years)*				
Mean (sd, min-max)	42.3 (15.6, 19–89)	42.6 (15.2, 18–78)	—	0.19
*Sex, n (%)*				
Female	62 (69.7)	74 (83.1)	136 (76.4)	0.034
Male	27 (30.3)	15 (16.9)	42 (23.6)	—
*ETT size in mm, n (%)*				
6.5	7 (7.9)	9 (10.1)	16 (8.99)	—
7.0	38 (42.7)	34 (38.2)	72 (40.45)	—
7.5	33 (37)	45 (50.6)	78 (43.82)	0.028
8.0	6 (6.7)	1 (1.1)	7 (3.93)	—
8.5	5 (5.7)	0 (0)	5 (2.81)	—
*Duration of intubation in hrs, n (%)*				
≤1	28 (31.2)	17 (19.1)	45 (25.3)	—
1-2	42 (47.2)	46 (51.7)	88 (49.4)	—
>2	19 (21.6)	26 (29.2)	45 (25.3)	0.14
*Provider level, n (%)*				
^∗^Resident	28 (31.5)	72 (80.9)	100 (56.2)	—
^∗∗^AO	51 (57.3)	10 (11.2)	61 (34.3)	—
AO students	2 (2.2)	3 (3.4)	5 (2.8)	—
Anesthesiologist	8 (9.0)	4 (4.5)	12 (6.7)	—

^∗^Anesthesiology resident, ^∗∗^anesthetic officer.

**Table 2:** ETT cuff pressure estimation by the PBP and LOR methods.

Cuff pressure ranges, *N* (%)						
Method	<20	20–30	31–40	>40	Mean (sd)	Proportion adjusted
PBP	8	20	18	43	51 (28.9)	51 (57.3)
LOR	11	59	18	1	27 (5.8)	12 (13.5)

All those <20 and >40 were adjusted by the manometer.

**Table 3:** Incidence of postextubation airway complaints in the study population.

Complaint, *n* (%)	All participants, *N* = 178	20–30 cmH_2_O, *N* = 98	31–40 cmH_2_O, *N* = 80	*p* value
At least one	113 (63.5)	56 (57.1)	57 (71.2)	0.052
None	65 (36.5)	42 (42.9)	23 (28.8)	—
*With cough*				
Yes	45 (25.3)	17 (17.3)	28 (35.0)	0.07
No	133 (74.7)	81 (82.7)	52 (65.0)	—
*With dysphagia*				
Yes	14 (8.0)	8 (8.2)	6 (7.5)	0.87
No	164 (92.0)	90 (91.8)	74 (92.5)	—
*With dysphonia*				
Yes	16 (9.0)	14 (14.3)	2 (2.5)	0.029
No	162 (91.0)	84 (85.7)	78 (97.5)	—
*With sore throat*				
Yes	89 (50.0)	42 (42.9)	47 (58.7)	0.035
No	89 (50.0)	56 (57.1)	33 (41.3)	—

## Abbreviations

ETT: Endotracheal tube
PBP: Pilot balloon palpation
LOR: Loss of resistance
ASA: American Society of Anesthesiologists.

## Data Access

The datasets analyzed during the current study are available from the corresponding author on reasonable request.

## Supplementary Materials

Supplementary Materials
*CONSORT 2010 checklist*. Endotracheal tube cuff pressure: a randomized control study comparing loss of resistance syringe to pilot balloon palpation.
